# Remission of a case of multiple Hymenoptera stings‐associated chronic urticaria during venom immunotherapy

**DOI:** 10.1002/ccr3.4188

**Published:** 2021-05-19

**Authors:** Marco Dubini, Valerio Pravettoni, Federica Rivolta, Giulia Segatto, Riccardo Asero, Nicola Montano

**Affiliations:** ^1^ Allergy and Clinical Immunology Residency University of Milan Milan Italy; ^2^ General Medicine, Immunology and Allergology Unit IRCCS Foundation Ca' Granda Ospedale Maggiore Policlinico Milan Italy; ^3^ Ambulatorio di Allergologia Clinica San Carlo Milan Italy

**Keywords:** chronic urticaria, Hymenoptera venom allergy, immunotherapy, venom allergy, venom immunotherapy

## Abstract

Hymenoptera stings mostly cause acute urticaria but we describe a case of CU after wasp stings which remitted during venom immunotherapy. IgE‐mechanisms have not been fully clarified in CU, except for isolated circumstances. In our case immunotherapy has played a positive role reducing immune cells reactivity and improving urticaria symptoms.

## Graphical abstract

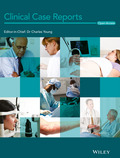

## 1 INTRODUCTION

The role of Immunotherapy in Chronic Urticaria is unclear, except for isolated circumstances. Hymenoptera sting causes acute urticaria and no report of CU after Hymenoptera sting can be found in the literature. We describe a case of onset of CU after multiple wasp stings that remitted during venom immunotherapy.

Urticaria is a clinical condition characterized by the development of angioedema, wheals (hives), or both. Urticaria affects about 15%‐20% of the population once or more during a lifetime, and 0.5%‐1.0% suffer from CU. Urticaria classification is based on its duration: Acute urticaria is distinct by the spontaneous occurrence of wheals, angioedema, or both for less than 6 weeks, while chronic urticaria (CU) is defined as the appearance of the symptoms for more than 6 weeks. CU is classified in different subtypes: chronic spontaneous urticaria (CSU) and inducible urticaria, with a very wide spectrum of clinical manifestations between these subtypes. Additionally, two or more different subtypes of urticaria can coexist in any given patient. Inducible urticaria comprises symptomatic dermographism, cold urticaria, delayed pressure urticaria, solar urticaria, heat urticaria, vibratory angioedema, cholinergic urticaria, contact urticaria, and aquagenic urticaria. CSU can also be due to unknown or known causes, for example, autoreactivity, that is, the presence of mast cell‐activating autoantibodies.

Urticaria is a disease driven by mast cells: Activated dermal mast cells release histamine, platelet‐activating factor, other mediators, and cytokines, but the pathogenesis is complex and has many additional features. In urticaria, the mast cell‐activating signals are heterogeneous and ill‐defined. There is a long list of both exogenous and internal causes of urticaria, including autoimmunity, infections, and allergic disorders. In the past decades, many advances have been made in identifying causes of different types and subtypes of urticaria. Among others, autoimmunity mediated by functional autoantibodies directed against the high‐affinity IgE receptor or IgE‐autoantibodies to auto‐antigens, pseudo‐allergy (nonallergic hypersensitivity reactions) to foods or drugs, and acute or chronic infections (eg, *Helicobacter pylori* or *Anisakis simplex*) have been described as causes of CU.

Anyway so far, IgE‐mediated mechanisms and the relevance of external allergens in CU have not been fully clarified. There remains little evidence that immunotherapy plays a role in CU other than in unusual circumstances. ^1^

## 2 CASE REPORT

We report a case of 34‐year‐old man with CU that appeared after a grade I Mueller reaction to Hymenoptera sting and remitted during venom immunotherapy (VIT), after months of unsuccessful therapy for CU.

He had a negative history of allergic and autoimmune diseases, and previous anaphylactic episodes, only a familiar history of intolerance to Aspirin and a personal history of gastroesophageal reflux disease, not requiring any therapy at that moment. He referred the first sting at the age of 20 by an unidentified insect and experienced a normal local reaction (smaller than 10 cm diameter). Afterward, about 1 year before our examination, he was stung by a vespid and again experienced a normal local reaction. Two months later, about 20 minutes after receiving multiple stings by a wasp swarm, itching hives developed all over his body. At the local hospital, intravenous methylprednisolone acetate and intramuscular chlorpheniramine were administered, with complete regression in 24 hours, and oral antihistamine for a week was prescribed. The day after, though, he manifested satellite lymphadenitis, which regressed in 5 days with steroid cream. Three weeks later, migrant generalized urticarial lesions started, showing spontaneous short‐lived wheals not in relation to foods or drugs, physical agents, or exercise. At our facility, routine blood tests were normal, except for mild leucopenia, which was already known before urticaria occurred; basophil and eosinophil counts were normal. Other causes of CU, such as acute or chronic infections and thyroid gland disorders, were excluded. Because of the prevalence of infections in our region, we ruled out the presence of *Helicobacter pylori* fecal antigen and beta‐hemolytic streptococci from throat and nasal swabs. Subtle urinary tract infection, including *Chlamydia spp*. and *Mycoplasma spp*., was excluded. Acute or misunderstood chronic viral infections, such as hepatitis (A, B, and C), Epstein‐Barr virus, cytomegalovirus, and other herpesviruses, were investigated. Antibodies against thyroid peroxidase, thyroid‐stimulating hormone receptor, anti‐thyroglobulin, anti‐endomysial, and anti‐tissue transglutaminase resulted negative. Plasma levels of D‐Dimer and acute phase reactants (erythrocyte sedimentation rate, C‐reactive protein, fibrinogen, ferritin) were normal. The levels of complement components excluded complement consumption or deficiencies. Autologous serum skin test excluded the presence of functional autoantibodies. Skin tests with common inhalant and food allergens (Lofarma), *Apis mellifera (ALK*‐Abelló, Denmark*),* and *Polistes dominula* (Anallergo) venoms were negative. Vespinae venoms tested positive, both *Vespula spp* (*ALK*‐Abelló) and *Vespa crabro* (Anallergo), at concentration of 0.1 μg/mL. Specific IgE tested positive for vespid venoms, respectively, 5.46 kU/L for *Vespula spp*, 0.47 kU/L *V crabro*, 0.69 kU/L *P dominula*. Analysis of the venom‐specific component revealed rVes v 5 (6.17 kU/L), rVes v 1 (< 0.10 kU/L), and rPol d 5 (1.07 kU/L). Total IgE level was 132 kU/L and serum baseline tryptase 7 mcg/mL. Notably, after skin tests with vespid venoms the patient suffered from an exacerbation of urticaria on his hips and thighs. Baseline assessment of disease activity with the urticaria activity score (UAS) showed a scarce control with oral antihistamine. Then, he started oral prednisone 25 mg/day, reaching the daily lowest effective dose of 5 mg. After 3 months, CU symptoms partially improved, and he agreed to start VIT because of the impairment of his quality of life and the close beginning of wasp season. Venom‐specific immunotherapy was started with *Vespula spp* extract (*ALK*‐Abelló) according to a 6‐week cluster schedule with weekly incremental doses of venom extract subcutaneously, until a maintenance dose of 100 000 standard quality units (SQ‐U/mL) was reached. During the up‐dosing phase, the patient remained under antihistamines therapy, because urticaria was still present. The achieved maintenance dose (100 000 SQ‐U/mL) was then given every 4‐6 weeks. During the up‐dosing phase and maintenance treatment, minor side effects such as local flushing and mild itching were reported and tolerated, and a worsening of the urticarial lesions occurred the day after each VIT injections. Nevertheless, after few months of VIT both pruritus and wheals improved gradually, and after 10 months, urticaria suddenly remitted and antihistamines were stopped. At present, CU symptoms have not recurred and the patient continues VIT (almost 5 years so far).

## 3 DISCUSSION

The diagnostic workup of CU includes among the major aims to exclude differential diagnoses.^1^ In fact, wheals or angioedema can be present in some other conditions, too.

Our patient never showed angioedema. This main feature allowed us to exclude diseases and syndromes defined by episodes of angioedema without hives, such as nonmast cell mediator‐mediated angioedema (eg, bradykinin‐mediated angioedema) and Gleich's syndrome (episodic angioedema with eosinophilia).

To distinguish different pathophysiologic mechanisms underneath the appearance of wheals is important to observe their cutaneous characteristics, their duration, and the presence of itching or burning sensation. As in our case, wheal in patients with urticaria has three typical features: a central swelling surrounded by reflex erythema, a fleeting nature lasting less than 24 hours, and association with itch. Therefore, we excluded the maculopapular cutaneous mastocytosis (urticaria pigmentosa) because of its peculiar dark‐colored skin lesions with a duration more than 24 hours, and the prebullous stage of bullous pemphigoid because of the absence of the typical vesicular eruption. In patients who display only wheals (but no angioedema), urticarial vasculitis, and auto‐inflammatory disorders, such as Schnitzler's syndrome or cryopyrin‐associated periodic syndromes (CAPS), need to be ruled out. Hives of autoimmune and auto‐inflammatory disorders have the typical feature of lasting more than 24 hours. Additionally, besides urticarial rash, CAPS (ie, familial cold auto‐inflammatory syndrome, Muckle‐Wells syndrome, or neonatal‐onset multisystem inflammatory disease) are characterized by recurrent fever attacks, arthralgia or arthritis, eye inflammation, fatigue and headaches, and Schnitzler's syndrome is characterized by recurrent fever attacks, bone and muscle pain, arthralgia or arthritis and lymphadenopathy and monoclonal gammopathy.

Serum sickness and serum sickness‐like reactions have been suspected because they can be triggered by insect stings and present with pruritic urticarial‐type lesions and lymphadenopathy without involvement of the mucous membranes. Symptoms appear days after the sting, overlapping a delayed reaction. We excluded these disorders because, unlike urticaria, the lesions often persist for days in the same area, and arthralgia or arthritis and fever are associated. Additionally, the long‐lasting skin lesions and the little improvement with steroid therapy are featured more typical of CU rather than serum sickness and serum sickness‐like reactions.^2^

The hypothesis behind urticaria progression in our case can be explained from several perspectives. First, vespid sting and urticaria onset could be coincidental and CU remitted spontaneously with VIT exerting no effect on its course. In fact, in most cases, CU is a self‐limiting disorder, persisting for 2‐5 years.^3^ Moreover, Hymenoptera sting causes acute urticaria, and in the literature, no report of CU after Hymenoptera sting can be found.

Alternatively, CU progression, from onset to remission, might be associated with some specific effects of venom allergens, from sting to VIT. Supporting this hypothesis, we observed a clear‐cut association between exposure to venom allergens and urticaria symptoms. In fact, type I allergy may be causative in a small number of CU patients and specific immunotherapy with these allergens may be beneficial in those patients.^4^ Examples of CU treated with allergen immunotherapy (AIT) can be found in the literature. Immunotherapy with sweat extract has been reported to be successful in the treatment of cholinergic urticaria, presumably due to the induction of tolerance to endogenous allergens.^5^ Sublingual immunotherapy with latex extract showed efficacy in patients with latex‐induced urticaria.^6^ A case of seasonal urticaria due to grass pollinosis was treated successfully with desensitization.^7^ House dust mite allergy has been hypothesized as a pathogenic factor in CU, and some patients have benefited from AIT.^8^ A study reported an improvement in CU symptoms using AIT with Giardia's antigen preparation.^9^ Finally, a review of the literature evaluated the efficacy and safety of AIT versus conventional therapy in the treatment of CU with positive results.^4^ There are no data about efficacy of VIT in CU.

Moreover, besides its specific effects, AIT exerts some nonspecific effects. AIT changes basophil and mast cell homeostasis and reduces the skin sensitivity not only to specific allergens, but also to histamine and nonspecific mast cell stimuli. Immunologic changes induced during the course of AIT include reduced mast cell and basophil activity and degranulation. After a few months, AIT produces a decrease in tissue mast cell, both connective and mucosal, in innate type 2 lymphocytes and eosinophil counts as well as in the release of their mediators.^10^, ^11^ As a matter of fact, reduced immune cells reactivity could contribute significantly to the improvement of urticaria symptoms, even if they have not been related to venom allergens. Therefore, in our patient VIT might have played a positive role, increasing the sensitivity threshold to nonspecific stimuli.

## 4 CONCLUSION

This is the first report in the literature of onset of chronic urticaria after Hymenoptera sting. Due to the need to undertake immunotherapy to protect the patient from venom allergy, a clear‐cut association between exposure to venom allergens and urticaria symptoms was revealed. In fact, IgE‐mediated allergy is a rare cause of CU, but should be considered in patients with intermittent symptoms. Additionally, besides its specific effects, immunotherapy exerts nonspecific effects on basophil and mast cell homeostasis. In our case, CU progression, from onset to remission, might be associated with some specific effects of venom allergens, from sting to allergen‐specific immunotherapy. This is the first report in the literature of a remission of chronic urticaria during venom immunotherapy.

## CONFLICT OF INTEREST

The authors certify that there is no conflict interest with any financial organization regarding the material discussed in the manuscript.

## AUTHOR CONTRIBUTIONS

VP and FR: provided the patient data regarding the allergic disease; MD and VP; wrote the manuscript; RA and GS: made contributions to interpretation of data. All authors critically read and approved the final manuscript.

## ETHICAL APPROVAL

All procedures were in accordance with the ethical standards of the responsible committee on human experimentation (institutional and national) and with the Helsinki Declaration of 1975, as revised in 2013. Informed written consent was obtained from the patient.

## Data Availability

The data that support the findings of this study are available from the corresponding author upon reasonable request.
